# An Episode of Pseudothrombocytopenia during Pembrolizumab Therapy in NSCLC Patient

**DOI:** 10.1155/2020/4196178

**Published:** 2020-05-08

**Authors:** Kinga Krukowska, Robert Kieszko, Katarzyna Kurek, Izabela Chmielewska, Paweł Krawczyk, Janusz Milanowski

**Affiliations:** ^1^Department of Pneumonology, Oncology and Allergology, Medical University of Lublin, Poland; ^2^Genetics and Immunology Laboratory, GENIM LLC, Lublin, Poland

## Abstract

Immunotherapy with immune checkpoint inhibitors (ICI) is a new option of treatment in a growing range of neoplasms. In addition to an antitumor effect, ICI are associated with autoimmune reactions resulting in a wide spectrum of toxicities that have not been seen in patients receiving chemotherapy. In this article, we present a case of a patient with advanced lung adenocarcinoma who developed an EDTA-dependent pseudothrombocytopenia (PTCP) during pembrolizumab therapy. To the best of our knowledge, this is the first reported case of EDTA-dependent PTCP occurring during immunotherapy treatment of nonsmall lung cell cancer with ICI. The phenomenon of EDTA-dependent PTCP may prompt clinical decisions, as unnecessary transfusions or even exclusion from pembrolizumab therapy. Therefore, it is important to be aware of PTCP as a possible side effect of this therapy.

## 1. Introduction

Immunotherapy with immune checkpoint inhibitors targeting programmed cell death 1 receptor (PD-1), programmed death ligand 1 (PD-L1), or cytotoxic T lymphocyte-associated antigen 4 (CTLA-4) is a profitable cancer treatment strategy. It has demonstrated advantageous outcomes in clinical practice including regression of advanced tumors and improvement of overall survival. The mechanism of action of monoclonal anti-PD-1 antibodies, such as pembrolizumab and nivolumab, involves a selective inhibition of PD-1 receptor on lymphocytes. The atezolizumab and durvalumab block PD-L1 activity on tumor cells and infiltrating immune cells. Ipilimumab efficiently binds to CTLA-4 receptor on activated T cells. In consequence, coinhibitory signals are disturbed allowing the antitumor immune response to be restored. Immunotherapy might be used in monotherapy as well as in combination with chemotherapy or radiotherapy in several malignancies including advanced stage nonsmall cell lung carcinoma (NSCLC), melanoma, bladder cancer, renal cell carcinoma, and other types of cancer [1–4].

Although immune checkpoint inhibitors (ICI) therapy have shown clinical benefits and general good tolerance, the literature describes many cases of immune-related adverse events (irAEs), particularly in the treatment of NSCLC or melanoma patients [5, 6]. Fatigue is the most frequently mentioned irAE correlated to PD-1, PD-L1, or CTLA-4 blockade. ESMO (European Society of Medical Oncology) Clinical Practice Guidelines reports other clinically significant immune-related toxicities: skin-affected toxicity (rash, pruritus, and vitiligo), endocrinopathies (thyroid gland disorders, hypophysitis), hepatotoxicity, gastrointestinal toxicity (colitis, diarrhea, nausea, vomiting, abdominal pain), or pneumonitis. Apart from those mentioned above, neurological, renal, cardiac, or haematological toxicities rarely occur [7, 8].

Thrombocytopenia is a common entity in patients with malignant diseases, especially during systemic chemotherapy treatment. It is defined as a platelet count of less than 150 000 cells/*μ*L. When the platelet count falls to 10 000 cells/*μ*L spontaneous bleeding may occur increasing the risk of surgical complications [9]. It may be caused by a central mechanism, as a consequence of bone marrow infiltration or as a result of treatment toxicity, a peripheral mechanism as it occurs, in microangiopathic disorders as disseminated intravascular coagulation (DIC), vasculitis or hemolytic uremic syndrome (HUS) or both. [10–12]. During chemotherapy, the presence of 3rd grade (platelets 25 to 50 000 cells/*μ*L) or 4th grade of thrombocytopenia (platelets <25 000 cells/*μ*L) induces delays or discontinuation of the treatment [11]. In this situation, a careful diagnostic evaluation is required.

Pseudothrombocytopenia (PTCP) is a falsely decrease of platelet count in EDTA blood samples. It is often due to the occurrence of EDTA-dependent antiplatelet antibodies that bind to the platelet surface and cause platelet clumping *in vitro*. As this entity can be mistaken for a false diagnosis of thrombocytopenia, affecting therapeutic decisions, it has to be kept in mind in the differential diagnosis of a decreased platelet count in cancer patients [13]. Nevertheless, there are no reports of PTCP in association with cancer immunotherapy thus far. In this article, we are presenting a case of lung adenocarcinoma patients with EDTA-dependent pseudothrombocytopenia which was detected during first-line pembrolizumab therapy.

## 2. Case Presentation

**Figure 1:**
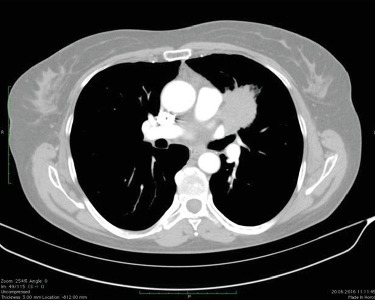
The CT scan showed new target lesion situated paravertebrally in the left lung.

Herein, we report a 58-years-old female patient with NSCLC. The patient was a former smoker with a ≥20 pack-year. The patient had not previously reported episodes of thrombocytopenia or bleeding episodes. The analysis of the whole blood test at the time of cancer diagnosis and also a previous blood test had not shown any alternations. In 2016, computed tomography (CT) scans of the chest demonstrated a tumor of the left lung (Figure 1). The bronchoscopic forceps biopsy of the mass in the bronchus of the left lung revealed adenocarcinoma. No *EGFR* mutations were detected. The patient underwent the upper lobectomy of the left lung in October 2016, and she was qualified to an adjuvant chemotherapy with cisplatin and vinorelbine in standard doses.

After third cycle of chemotherapy, the patient developed neutropenia (neutrophil count of 780 cells/*μ*L) and anemia (erythrocytes count of 3.35 million cells/*μ*L, haemoglobin concentration—10.5 mg/dL). The fourth cycle was temporarily deferred. She was treated with G-CSF with the improvement of neutrophils. After a few days, anemia also improved. The fourth cycle of chemotherapy was administered, and the first-line treatment ended up successfully.

**Figure 2:**
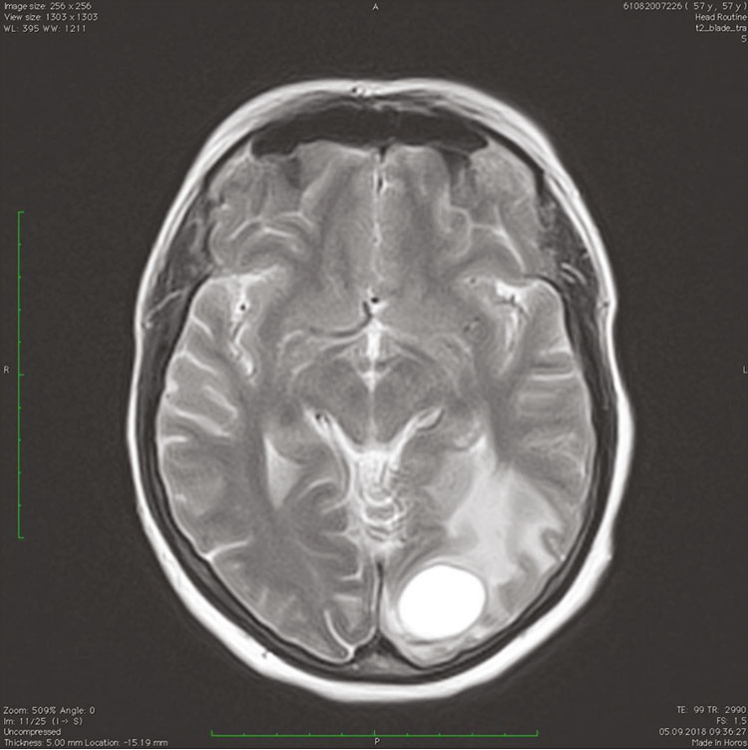
Brain metastatic lesion in left occipital lobe with dimensions of 2.5 cm × 2.1 cm × 2.9 cm.

After one year follow-up, cancer progression was detected in a control CT scan showing a mass in the top of the left lung with a diameter of 21 mm. In MRI and PET-CT, we observed metastatic lesions—two with a diameter of 19.3 mm in the left occipital lobe of the brain. In September 2018, the patient underwent stereotactic radiotherapy of brain metastasis with a positive response. PD-L1 expression in 70% of tumor cells was detected by immunohistochemistry, without expression of ALK protein. The patient was qualified for second-line treatment with immunotherapy after meeting inclusion criteria, including the reference level of blood cell count and biochemical test results, the presence of target tumor situated paravertebrally in the left lung (Figure 2) and no evidence of metastatic progression in the central nervous system.

In October 2018, pembrolizumab at a dose of 200 mg every three weeks was initiated. The patient tolerated four cycles of immunotherapy with no side effects. In the beginning of 2019, before the administration of the 5th cycle of pembrolizumab, a routine blood cell count test was performed, showing a sudden decrease in platelet count to 53 000 cells/*μ*L in EDTA-K_2_ sample. Unfortunately, the blood smear was not performed. Currently, it is impossible to reperform it as the blood sample has not been archived. However, to explain this phenomenon, two additional blood samples were drawn in sodium citrate anticoagulated probe and Thrombo-Exact (Sarstedt®) probe with magnesium salt. In both those samples, we found the platelet count at an acceptable level (223 000 cells/*μ*L). Testing two additional, differently anticoagulated blood samples were highly likely to make a possible diagnosis of PTCP in the EDTA-K_2_ sample. The initial suspicion of thrombocytopenia was excluded, and a 200 mg dose of immunotherapy was maintained.

Platelet count was strictly monitored during the treatment, but no further episode of thrombocytopenia occurred. After ninth pembrolizumab infusion, the CT scan demonstrated an enlarged metastatic lesion in the left occipital lobe. She underwent whole-brain radiotherapy. Immunotherapy was discontinued, and the patient was qualified for chemotherapy with cisplatin and pemetrexed. Currently, she is being treated with chemotherapy that is being well-tolerated.

## 3. Discussion

Pseudothrombocytopenia is a false decrease of the number of platelets that occurs in EDTA blood samples. It is mostly associated with the presence of EDTA-dependent antiplatelet antibodies that cause platelet agglutination *in vitro*. These antibodies are usually IgG, IgM, and IgA cold agglutinins. They bind to IIb/IIIa glycoprotein (GP) receptor on the platelet surface, triggering agglutination in the probe. In EDTA-coagulated samples, the decrease of temperature might activate a reaction since cold agglutinins react optimally at 4-20°C. To rule out this entity, the blood sample needs to be warmed or the test should be carried out in a probe with sodium citrate or magnesium salt [13, 14]. In this case, the platelet count was reevaluated for both anticoagulants.

The phenomenon of PTCP is very rare (it appears in 0.1% of the population), and it has been identified in various diseases and in healthy people. PTCP has been reported in infections, sepsis, autoimmune diseases, thrombotic or cardiovascular disorders and also in association with malignancies like bladder cancer or neuroendocrine cancers [15, 16]. PTCP has been observed during insulin, valproic acid, or antibiotic treatment [17, 18]. Balcik et al. studied 49 cases of PTCP diagnosed patients. The authors made a platelet assessment in EDTA and citrate-coagulated samples, assessing for the existence of cold agglutinins, concomitant diseases, medications, malignancy, pregnancy, and antinuclear (ANA) or anticardiolipin (ACA) autoantibodies. They found that hospitalization, infection, pregnancy, and the use of low molecular weight heparins were significant risk factors for PCTP. However, there was no significant relationship between PTCP presence of autoantibodies [19].

It is known that haematological immune-related events (haem-irARs) caused by immunotherapy with ICI may occur, even though it is not a common phenomenon. A comprehensive study made by Delanoy et al. in a group of 745 cancer patients receiving immunotherapy showed its frequency of 0.5%. The authors, using data from three French registries of irAEs, found 35 patients affected by haem-irARs including 12 (34%) patients with NSCLC. Nevertheless, among all types of immune-related haematological toxicities, the most common were neutropenia, thrombocytopenia, and autoimmune hemolytic anemia (AHA). Only three patients had AHA associated with the presence of cold agglutinins. There were other toxicities mentioned like pancytopenia or aplastic anemia, bicytopenia, and pure red cell aplasia [6].

In another study, Sui et al. analyzed and compared results of 26 clinical trials describing haematological toxicities during ICIs therapy in various cancer patients. In three trials, pembrolizumab therapy for NSCLC patients was administrated. The authors showed that anemia, thrombocytopenia, leukopenia, and neutropenia as toxicities among haem-irAE [20].

Several cases of haematological immune-related adverse events during ICI therapy have been reported. [21, 22]. Bangley et al. presented a case of thrombocytopenia induced by anti-PD-1 treatment in NSCLC patients. [23]. Cases of immune thrombocytopenia have been observed and described previously in melanoma patients treated with nivolumab, ipilimumab, or pembrolizumab. However, there were no cases of EDTA-dependent PTCP [24, 25].

To the best of our knowledge, this is the first reported case of EDTA-dependent PTCP in a patient with NSCLC treated with immune checkpoint inhibitors. Our case highlights the importance of an accurate and comprehensive diagnostic approach that must be kept in mind in this situation and it can occur during ICI treatment.
